# Progress of Veterinary Science

**Published:** 1883-07

**Authors:** 


### Progress of Veterinary Science.

A CASE OF TRANSMISSION OF TUBERCLE FROM THE HUMAN SPECIES TO THE DOMESTIC CAT.—In the culture of medical science in these days of true and rapid discussions, the question of the transmission of tubercle from animals to man is proposed. I take the liberty to narrate a fact referable to a diametrically opposite problem, namely; the transmission of tubercle from man to animals. The case was that of a cat, two years of age, which died tuberculous after having repeatedly eaten the expectorated matter from a lady who died soon after of phthisis. On my first visit, by request of the owner of the cat, the animal presented a cadveric appearance, with a physiognomy of suffering and starry and lusterless hair. For some time previous it had been noticed that it had lost its natural beauty and vigor, lost appetite and suffered from severe cough and other symptoms which threatened a fatal termination. The cat died and I made a post mortem examination which verified my diagnosis. It was true tubuculosis. On opening the thoracic cavity I found white nodules of irregular form disseminated throughout the entire substance of both lungs, isolated or confluent and varying in size from millet seed to a grain of corn. I found some of these tubercles on the costal pleura, some on the pericardium, and, note the circumstance, only one of the size of a grain of wheat on the right side of the heart near the apex. I did not have time to examine these nodules microscopically. Examined thus superficially I found variation in their consistency, some hard and calcarious, others soft and containing pus. Will these data be sufficient toprove the cat tuberculous? I believe so.—G. BREZZO in *Il medico Veterinariio, Torino.*

TUBERCULOSIS AND HYDROTHORAX IN A FEMALE RHINOCEROS.—" 'Mongo' a female rhinoceros, about sixteen years of age, weighing about five thousand pounds, had been for the last thirteen years one of the attractions of Mr. P. T. Barnum's show. Ever since she joined the show she had enjoyed apparently good health, and at the time of her arrival in New York on the morning of last Wedesday, seemed to be as well as usual. She was on the evening of that day fed as usual, and when on Thursday morning her cage was opened, she was found dead, lying in her natural position, and showing that she had died without a struggle. Her cadaver was brought to the American Veterinary College, when a *post-mortem* examination was made.

" An incision was made in the median line of the body from the chest to the pubes, and the skin, more than an inch thick, was dissected from the body. The abdominal cavity being opened and the contents removed, the following principal lesions were found: Near the stomach and between the cardiac and pyloric opening of that organ, was found a large, nodulated tumor, resting upon the small curvature of the stomach, tuberculous in character, and filling the entire space left between the two openings of that curvature. Spleen and the liver presented several nodulated tuberculous deposits much smaller than the first one. Near the quadrifurcation of abdominal aorta a number of tuberculous deposits were also found. The uterus is mammilated here and there on the body and the horns with tuberculous masses of the size of a walnut, situated in the cellular tissue between the muscular and mucous coat.

"All the organs of that cavity are healthy—the stomach, the intestines, the kidneys, the suprarenal capsule—with the exception of the small deposits already alluded to in the liver and spleen.

"On opening the thoracic cavity it was found to contain twenty-eight gallons of clear serous fluid, which had crowded the lungs into the upper portion of the chest, and the anterior lobes of both lungs were adherent to the thoracic walls by strong fibrous bands. The pleura presented a number of thickened spots containing tubercular deposits. The mediastinum was filled with tuberculous masses, varying in size from that of a pea to that of a child's head. The lungs were dark, of a bluish color, slightly emphysematous at their lower border, and with their surfaces filled with miliary tubercles, very heavy and dense. Heart has both cavities empty and presented tuberculous granules on the tricuspid and semilunar valves. At the base of the heart, around the aorta and pulmonary artery, was found a large mass of tubercles. The pericardium is adherent to the pleura and to the lungs by fibrous bands, but is otherwise healthy. The pericardiac and cardiac lymphatic ganglions are enormously enlarged and indurated. On section they presented a circular wall of dense fibrous tissue, sending off shoots into the centre of the mass, and interlacing each other formed a kind of reticulum or stroma, with a dark greenish aspect in the meshes, containing tubercular deposits of various sizes. On the superior face of the sternum three large tubercles were found attached, and the axillary glands of both sides were very much enlarged, dense, of whitish color. The pectoral, prepectoral, and lymphatic glands of the neck were also much enlarged, indurated, and tuberculous in their character.

"Both of these affections were evidently of old standing, and readily accounted for the sudden death of the animal."—*Medical Record.*

SCARLATINA AMONG HORSES.—In Williams' work on Veterinary Medicine, republished by Wm. Wood & Co., 1879, an extended but somewhat confused account is given of equine scarlatina. In Fleming's "Animal Plagues," p. 482, we read: "In 1775 an inflammation of the pituitary membrane and catarrhal angima prevailed among horses and dogs, which were affected *before* mankind. * * * In 1776 an epizootic catarrh among horses succeeded the epidemic influenza of man in the spring."

*Ibid*, p. 446. A gangrenous sore throat prevailed in St. Domingo in 1772. The pharynx, œsophagus and trachea were covered with aphthæ in cows in France. In Russia and Wallachia, in an epizooty, the eyes were inflammed and projecting; the glands of the neck and even of the head were swollen, and in other cases the axillary or inguinal glands were involved. There was a discharge of pus from the nostrils. The disease was so contagious that if one was affected all others were seized in a very short space of time. Men, however, were not affected, though they handled the sick animals.

*Ibid*, p. 440. Gangrenous sore throat prevailed in Tartary in 1770, attacking men, horses, and cows. The disease showed itself, all at once, in the healthiest subjects of all ages and either sex. At first the skin became slightly reddened, then broke out into pimples, usually on the face of man and the flank and bellies of animals. It may have been small-pox, but was supposed to arise from the stings of venomous insects. Still the animals usually died, and the mortality was very great.

*Ibid*, p. 434. In 1770 a contagious epizooty appeared in France. It was apparently gangrene of the throat, but differed from the epidemic of 1762. The disease concentrated itself chiefly to the nasal cavities and upper parts of the respiratory passages. The eyes were suffused and the conjunctiva inflamed; the pharynx and nasal cavities also. The parts at the back of the mouth were highly inflamed, and yellow matter flowed from the nostrils, * * * and the nasal discharge became thin, fetid, and bloody.

*Ibid*, p. 425. "In 1767 scarlatina was very prevalent in France, and puerperal fever in Normandy. P. 426. In France the lower animals suffered equally with mankind. P. 427. So virulent was the contagion that, from the moment a horse was attacked in a stable, not only were the others quick in receiving it, but the infection lingered in it for many weeks afterwards, and was not slow in attacking horses brought there during this time, so that it was necessary to leave these stables empty for a long period in order to have them well aired and disinfected, and also whitewashed repeatedly, so obstinately would this malady leave traces of its fury and subtilty in the walls."

*Ibid*, 423. In 1764 a so-called aphthous disease affected horses, cattle, sheep and pigs. They had great heat in the head, and began to run from the eyes; the nose and mouth swelled, especially the palate; * * * saliva flowed in great quantities, and when the disease assumed a serious aspect it choked them, or made them appear as if they had something in their throats, etc.

*Ibid*, p. 421. Apthous fever and erysipelas appeared as epizootics, attacking horses as well as cattle. * * * The men who were affected by the disease experienced great difficulty, amounting to an impossibility sometimes, in swallowing, owing to inflammation and tumefaction in the back part of the throat. "Dr. Nicolau thought the disease arose in marshes, which in summer are crowded with flocks and herds, the putrid exhalations from which, engendered by the heat of the sun, gave rise to disease in animals, and putrid fevers among human beings." P. 418.

A. D. 1762. *Ibid*, p. 415. An epidemic of gangrenous sore throat (angina gangrenosa) in France affected cattle, horses and mules, and was very destructive. Bougelat, the founder of the French veterinary schools, was sent to investigate it. He found painful swellings in the region of the lower jaw, and along the neck; discharge from the nose; gangrene in the back part of the throat, pharynx and larynx, and in the cellular tissue enveloping or separating these parts; and in the œsophagus and trachea the membrane enveloping the base of the soft palate was black, livid, and covered with ulcers, which had gnawed away the base of the tonge. Is not this diphtheria ?

The external tumefaction extended from the neck to all the inter maxillary glands, the neck and bronchiæ forming considerable external tumors.

P. 416. The throat was red, brown, and sometimes black.

The causes were drought, heat, bad herbage, and more particularly the extremely unhealthy, stagnant water the animals were compelled to drink. This disorder was contagious.—*Sanitarian.*

Anthrax in the Hog somewhat different from the common form.—In the autumn of 1882 a fatal sickness attacked the swine of Betolle and other parts of Valdichiana, decimating entire herds.

The disease attacked by preference the young animals not sparing, however, the adult and older ones. It was considered contagious because as one became attacked several others of the herd met the same fate.

As to the symptoms of the disease the animal became depressed, lost appetite, and fever set in. Towards the end the patient maintained nearly always the recumbent position, the skin becoming reddened in those parts which lacked pigment, without, however, presenting any sign of eruption. Generally a period of erethism followed during which the animal jumped and shook itself. Death took place on the fourth or sixth day from the commencement of the attack.

Recovery was rare, the period of erethism not taking place in these cases, and the animal becoming lively, recovering apetite, etc. The diagnosis being uncertain from symptom alone, I made several post-mortem examinations, often accompanied by Francesco Marchi, V. S. The result of which I will not describe in detail but restrict myself to refer to the general characters.

In all the cases the prevailing characters were severe gastritis often accompanied by partial enteritis limited generally to the duodenum. The mucous membrane of the stomach was of a deep red color, swollen and softened, in the neighborhood of the pylorus, rarely near the cardiac orifice. The duodenal mucous membrane was in many places abundantly infiltrated with biliverdine. Evolution of gas took place in the stomach and intestines, (meteorism). In only one case I found gangrene of the gastric parietes. In some cases an intense unilateral pueumonia with hepetization: in others the inflammatory action was confined to one lobe. The parenchyma of the lung presented a dark red color, black blood exuding on section, the surface of the wound having a gran-granulous appearance. In many cases the pericardium contained a small quantity of yellowish fluid in which white flakes were perceived. Sometimes the kidneys were found hypertrophied with their parenchyma softened.

The spleen was always in a normal state, not softened as usually takes place in anthrax. The liver often presented a physiological state, sometimes hypertrophied at other times full of black and uncoagulated blood. The blood was in some cases found in a normal, in others in an imperfect state of coagulation.

The fact that the spleen and often the liver were neither hypertrophied nor softened, the easy coagulation of the blood observed in many cases and the absence of some of the other anatomico-pathological characters peculiar to anthrax made me doubt this to be the disease under consideration; but before making a new diagnosis I wished to examine microscopically the blood of the animals in question. On doing so I found the characteristic germ, *Bacillus anthracis.* Repeated examinations always gave the same results.

As regards the therapeutic means prescribed, I found, besides scrupulous cleanliness of the stables, isolation of the sick, and the combustion or or deep burial of the carcasses, the sulphite of sodium to have a good effect, especially if given as a preventive. By these means I obtained through the assistance of Sig. Marchi, exemption from the disease of many pigs and even cure in many of the non-malignant cases.

N. PASSERINI, in *Gironale di Anat. Fisiol. Patol. degli animali.—Pisa.*

IMMUNITY OF ANIMALS FROM SYPHILITIC INOCULATION.—Professor Neumann has made a number of attempts to inoculate animals with syphilis, but without success. The experiments were made with the greatest care, the virus being taken directly from the diseased person and introduced into the body of the animal. The animals experimented upon were kept under observation for a considerable period of time after the inoculation. In no case did any results obtain other than those which would naturally follow the introduction of an irritating material into the tissues. Nothing that bore any resemblance to a chancrous tumor was observed. The animals employed in these experiments were three apes, three rabbits, a horse, a hare, a white rat, a marten, and a cat. The total number of inoculations was fifty-four. Neumann concludes from these experiments that we must regard syphilis as distinctly a disease of man.—*Med. Central-Zeitung.*

THE REPARATIVE PROCESS IN CARTILAGE.—Dr. Gies opened the knee-joint in young dogs under strict antiseptic precautions, and excised a piece of the articular cartilage, taking care not to wound the bone beneath. The operation was never followed by the slightest inflammatory reaction. After the animals were killed the wound in the cartilage was seen to be surrounded by an "atrophic zone," in which the cells were degenerated or had disappeared. Around this was a "proliferating zone," characterized by enlargement and segmentation of the cartilage cells and an increase in the number of nuclei. In no instance was there the least trace of a reparative process. Incised wounds of the cartilage, even after five months, showed no tendency to union, Here also, as in the case of actual loss of tissue, were seen the athropic and hypertrophic zones parallel to the fissure. In a second series of experiments, the knife with which the cartilage was incised was previously dipped in a putrid infusion. The results now obtained were the same as those of other experimenters who operated without antiseptic precautions. Proliferations from the inflamed synovial membrane filled the wound in the cartilage with round and spindle cells. Presently cartilage cells appeared in the newly formed tissue, and in three months the wound was entirely filled with hyaline cartilage, so that scarcely any trace of the former injury could be discerned. —*Centralblatt für Chirurgie.*

TO SETTLE THE QUESTION whether or not it is possible for ova to travel across the peritoneal cavity or that of the uterus, Dr. Leopold, of Leipsig, has performed some important experiments. In these he made use of eight rabbits. In each case he opened the abdomen, tied the right Fallopian tube in two places and cut out the piece between the ligatures; the left ovary was carefully removed, then the abdominal wound was closed. After thorough recovery each animal was put to the male. In six cases the result was entirely negative, but in two pregnancy followed. The abdomen of the latter was opened; in one, four placentæ were found in the left horn of the uterus, and one in the right. He thinks these experiments settle the question. In these rabbits ova could not reach the uterus by travelling across the peritoneum from the right ovary to the left Fallopian tube; and only could get into the right horn of the uterus by passing down the left horn and up the right. They prove, therefore, that it is possible for ova to migrate, not only across the peritoneum, but across the uterine cavity.—*Weekly Medical Review.*

EXAMINATION OF A HIBERNATING SNAKE.—On the 14th day of February, 1883, one of my neighbors, while digging among some rocks near this place, suddenly came upon the winter quarters of a small "bull snake." The serpent was in a torpid state from the cold weather and was easily killed. During the afternoon of the 15th I happened to be at the place where it was killed, and procured it for examination. It measured 3½ feet in length, and weighed 7 ounces. On opening the body, the stomach was found to be entirely empty; the bowels were almost empty, containing only a small amount of creamy looking substance in the lower bowel, streaked and tinged with something greenish resembling bile. Being a female, the parts answering to ovaries were large and congested, and each consisted of 26 distinct parts or divisions. The liver was very small and pale. The gall-bladder was large and full of bile, containing nearly half a fluid drachm. The lungs were fully inflated and extended on each side of the spinal column nearly the whole of the middle third of the body. The heart and arteries were nearly full of bright red blood. The spleen was 7 inches long, and weighed one-fourth of an ounce, which it will be seen was one twenty-eighth of the weight of the entire animal. It was of a dark red color and seemed to be rich in blood.

Is it probable that this enormous spleen serves as a reservoir of nourishment upon which the animal lives after its stock of adipose tissue is gone? The cavity of the body contained but very little fat, perhaps not more than 10 grains. Snakes at this altitude usually go into winter quarters about the 20th of October, and emerge from their winter homes about the last of April, or 15 days earlier if the weather be warm. This gives them a period of about six months in which they take no food. If not for the purpose of nourishing the body during this long fasting period, what can be the use of such an enormous spleen?—*Pacific Medical and Surgical Journal.*

CONSUMPTION AND TUBERCULOSIS.—In a lecture delivered before the East Berwickshire Agricultural Association, on Saturday week, Dr. Fleming remarked, that the disease called consumption in man, and tuberculosis in animals, was of the greatest possible moment to the whole world, because now known to be communicable, both by ingestion and cohabitation. If the milk and flesh of a consumptive animal were given to a healthy animal, they produce consumption in that animal. This question was assuming such importance that the European governments were taking active measures to ascertain everything regarding it. The pig, in its organization, was next to our own species, and they could produce consumption in that animal. Therefore the germs of tuberculosis possessed for them an immense interest and importance. If they could trace the origin of the disease producing tuberculosis in animals and consumption in human beings, then at once they had the key to its prevention, and they could stamp out the disease. He believed that our own government would have to include this disease, tuberculosis, under the Contagious Diseases (Animals) Act, and stamp it out. The dairies of our large towns fostered and spread the disease, one affected animal being sent to the butcher, and another in its place being put into the infected stall. Five per cent. of our dairy stock were infected with this disease of tuberculosis. The better bred the cattle were, the more susceptible were they of tuberculosis. He was afraid the disease was on the increase, and until the fact was recognized that it was an infectious disease, they would be unable to check the malady, and the terrible scourge of the bovine and human species would remain and spread.

